# Non-antibiotic treatments for bacterial diseases in an era of progressive antibiotic resistance

**DOI:** 10.1186/s13054-016-1549-1

**Published:** 2016-12-16

**Authors:** Steven M. Opal

**Affiliations:** 1 Infectious Disease Division, Alpert Medical School of Brown University, Providence, RI USA; 2 Ocean State Clinical Coordinating Center, 1 Virginia Ave, Suite 105, Providence, RI 02905 USA

**Keywords:** Antibiotic resistance, Novel therapies for bacterial infections, Quorum-sensing inhibitors, Phage therapy, Monoclonal antibodies to treat bacterial infections

## Abstract

The emergence of multi-drug resistant (MDR) microbial pathogens threatens the very foundation upon which standard antibacterial chemotherapy is based. We must consider non-antibiotic solutions to manage invasive bacterial infections. Transition from antibiotics to non-traditional treatments poses real clinical challenges that will not be easy to solve. Antibiotics will continue to reliably treat some infections (e.g., group A streptococci and *Treponema pallidum*) but will likely need adjuvant therapies or will need to be replaced for many bacterial infections in the future.

Recent discoveries of plasmid-transferable genes that mediate resistance to carbapenems [1] and colistin [2] indicate that the last defensive wall against multi-drug resistant (MDR) pathogens has already been breached. We now face the uncomfortable reality that the post-antibiotic era has arrived when dealing with pan-resistant bacterial pathogens. We need to consider our remaining options and develop new ones in a world where antibiotics can no longer be counted upon to cure infections. Non-antibiotic opportunities to treat serious bacterial infections exist as possible options (Table 1).

**Table 1:** Summary of some non-antibiotic inhibitors of bacterial growth and/or pathogenesis

Treatment strategy	Mechanism of action	Possible benefits
Hemoperfusion devices [3, 4]	Extracorporeal filters that clear blood pathogens by their physiochemical properties	Quickly reduce blood concentrations of selected bacteria by orders of magnitude
Quorum sensing inhibitors [5, 6]	Disrupt intercellular signaling between bacteria to block coordinated tissue invasion	Blocks sensing of necessary concentrations of bacteria for optimal synthesis of virulence and invasion genes
Lytic bacteriophage [7, 8]	Bacteriolysis induced by selected lytic phage or phage cocktails	Parasitic predators of bacteria that can be used as highly specific, targeted, bactericidal agents
Polyclonal or monoclonal antibodies [9–11]; immune adjuvants [12]	Improved bacterial vaccines, transgenic cattle for polyclonal immunotherapy; designer monoclonal antibodies; immune-stimulant therapy for sepsis induced immunosuppression	Active or passive immunotherapy to opsonize bacteria or inhibit exotoxins and virulence factors; adjuvants to stimulate cellular immune function
Liposome-based cyto-toxin inhibitors [13]	Engineered liposomes to serve as cell membrane decoys to absorb bacterial cyto-toxins	Capture pore-forming cyto-toxins and protect host cell membranes from cellular injury
Non-immune toleralizing approaches [14, 15]	Treatments allowing the host to survive and compensate for pathogen presence or until immune clearance removes the pathogen	Permits the host to tolerate the pathogen until cleared by immune or non-immune mechanisms (e.g., oral or intravenous fluids for cholera)

## Hemofiltration devices

Extracorporeal pathogen removal filters are in development which can bind and remove an array of blood stream pathogens. Multiple device filters are being studied; two of the more interesting ones include the use of mannose binding lectins [3] or bound heparin [4]. Reduction in the bacterial load by hemofilters could theoretically allow the host innate and adaptive immune systems to remove residual pathogens despite pan-resistance to antimicrobial agents.

## Quorum sensing inhibitors

Many bacteria employ some form of intercellular communication to alert pathogens about their collective bacterial concentration. If high concentrations are detected, pathogens can switch their transcription profiles to an invasive phenotype [5, 6]. An impressive array of natural and synthetic molecules can block quorum sensing and improve outcomes in experimental models of systemic infection. Whether quorum sensing inhibitors will ever be of practical clinical benefit against MDR pathogens remains the subject of considerable debate [5, 6].

## Lytic bacteriophages

The use of bacteriophages (viruses that lyse specific bacteria) as a replacement for antimicrobial agents against MDR pathogens remains an attractive option despite numerous challenges [7, 8]. Phage therapy to treat bacterial infection was introduced in the early 1920s and is still in clinical use in some regions in Eastern Europe and in Georgia [8]. Phage therapy is now regaining widespread interest as antimicrobial resistance is reaching a global crisis.

Bacteriolysis by selected lytic phages is likened to the activity of a rapidly bactericidal antibiotic against susceptible bacteria. Phage invade bacteria via attachment to surface receptors on bacteria where they replicate intracellularly and kill the bacterial host by digesting the peptidoglycan cell wall. Phage are ubiquitous in nature and are harmlessly ingested in our diet by the millions each day [8]. Phage therapy can be administered topically on open wounds or surface infections [7] or given intravenously for use in systemic infections.

Despite all the theoretical advantages of phage therapy for MDR pathogens, numerous drawbacks and practical challenges exist. The major problem is their exquisite specificity. Phage infect only one strain of bacteria, thereby precluding their use as empiric therapy for acute infections. The causative bacterium responsible for the infection must be identified; then a suitable phage therapy can be fashioned from existing stocks of phage. Stocking a hospital laboratory with a complete library of phage for every conceivable bacterial pathogen will be a major challenge indeed [8].

## Advanced immunotherapies

Immunotherapy to treat infectious diseases is not a new idea, but innovations in the generation of high affinity, human polyclonal or monoclonal antibodies against an array of molecular targets makes this an attractive approach. Active immunizations with adjuvanted, multi-eptitope bacterial vaccines are in development, as are monoclonal and polyclonal antibodies, as passive therapies against bacterial pathogens [9–11]. Transchromosomic cattle have been developed that can deliver high volumes of high quality, human polyclonal antibodies against bacterial and viral antigens [11]. Monoclonal antibodies can be designed with advantageous features and half-lives that can opsonize bacteria or inhibit virulence factors without the need for antibiotics [9, 10].

Immune adjuvants are in clinical development to booster cellular and humoral adaptive immunity of the host [12]. A number of adjuvants are under investigation, including interleukin-7, granulocyte macrophage-colony stimulating factor, programmed cell death ligand-1 antibody, among other strategies. Such immune adjuvants could benefit patients with sepsis-induced immune suppression [12].

## Alternative efforts to limit virulence

Liposome-based cyto-toxin inhibitors [13] have been engineered to capture a variety of cell membrane lytic toxins produced by bacteria. These liposomes serve as cell membrane decoys to absorb cyto-toxins and thereby protect human cells from injury. This non-antibiotic defense mechanism is protective experimentally and could complement anti-microbial agents in treating exotoxin-producing bacterial infections.

## Non-immune tolerance to pathogens

Non-immune tolerizing events allow the host to survive and co-exist in the presence of a potential microbial threat. This represents a novel way of approaching the problem of MDR pathogens [14, 15]. Treatments would be aimed at allowing the host to compensate for pathogen presence until immune clearance removes the pathogen. An example of non-immune tolerance is seen in the differential susceptibility of mice (and likely humans) to Ebola virus disease [15]. Mouse strains vary dramatically in their susceptibility to the same strain and dose of Ebola virus. The genetic explanation is found primarily in the variation of expression of a single gene known as *Tek*, the human homologue for tyrosine kinase receptor for angiopoietin-1. High levels of angiopoietin1/Tie2 promote endothelial barrier protection. Ebola viruses specifically target the endothelium and kill endothelial cells. Mouse strains with high levels of Tek are better able to defend their endothelial surfaces until the adaptive immune cells (cytotoxic CD8 cells) arrive at about 7 days into infection to clear the virus. Perhaps non-immune therapeutics against infectious diseases might provide some options against MDR pathogens in clinical medicine.

## Summary

The progressive spread of antibiotic resistance genes is forcing us to reconsider our treatment options against some bacterial pathogens. Treating bacterial infections will likely become more challenging in the future. We need to protect the antibiotics we already have, develop new ones, and redouble our efforts to generate novel therapies against bacterial pathogens.
